# Cobalt Sulfide Confined in N-Doped Porous Branched Carbon Nanotubes for Lithium-Ion Batteries

**DOI:** 10.1007/s40820-019-0259-z

**Published:** 2019-03-29

**Authors:** Yongsheng Zhou, Yingchun Zhu, Bingshe Xu, Xueji Zhang, Khalid A. Al-Ghanim, Shahid Mahboob

**Affiliations:** 1 College of Chemistry and Materials Engineering, Anhui Science and Technology University, Bengbu, 233030 People’s Republic of China; 2 Key Laboratory of Inorganic Coating Materials CAS, Shanghai Institute of Ceramics, Chinese Academy of Sciences, Shanghai, 200050 People’s Republic of China; 3 Key Laboratory of Interface Science and Engineering in Advanced Materials, Ministry of Education, Taiyuan University of Technology, Taiyuan, 030024 People’s Republic of China; 4 Beijing Key Laboratory for Bioengineering and Sensing Technology, Research Center for Bioengineering and Sensing Technology, School of Chemistry and Bioengineering, University of Science and Technology Beijing, 30 Xueyuan Road, Haidian District, Beijing, 100083 People’s Republic of China; 5 Department of Zoology, College of Science, King Saud University, Riyadh, Saudi Arabia; 6 Department of Zoology, GC University, Faisalabad, Pakistan

**Keywords:** Lithium-ion batteries, Nitrogen doping, Cobalt sulfide, Branched carbon nanotubes

## Abstract

Lithium-ion batteries (LIBs) are considered new generation of large-scale energy-storage devices. However, LIBs suffer from a lack of desirable anode materials with excellent specific capacity and cycling stability. In this work, we design a novel hierarchical structure constructed by encapsulating cobalt sulfide nanowires within nitrogen-doped porous branched carbon nanotubes (NBNTs) for LIBs. The unique hierarchical Co_9_S_8_@NBNT electrode displayed a reversible specific capacity of 1310 mAh g^−1^ at a current density of 0.1 A g^−1^, and was able to maintain a stable reversible discharge capacity of 1109 mAh g^−1^ at a current density of 0.5 A g^−1^ with coulombic efficiency reaching almost 100% for 200 cycles. The excellent rate and cycling capabilities can be ascribed to the hierarchical porosity of the one-dimensional Co_9_S_8_@NBNT internetworks, the incorporation of nitrogen doping, and the carbon nanotube confinement of the active cobalt sulfide nanowires offering a proximate electron pathway for the isolated nanoparticles and shielding of the cobalt sulfide nanowires from pulverization over long cycling periods.

**Electronic supplementary material:**

The online version of this article (10.1007/s40820-019-0259-z) contains supplementary material, which is available to authorized users.

## Highlights

- A novel hierarchical structure constructed by encapsulating cobalt sulfide nanowires within nitrogen-doped porous branched carbon nanotubes (NBNTs) is designed for lithium-ion batteries.
- The unique hierarchical Co_9_S_8_@NBNT electrode displayed a reversible specific capacity of 1310 mAh g^−1^ at a current density of 0.1 A g^−1^.

**Electronic supplementary material:**

The online version of this article (10.1007/s40820-019-0259-z) contains supplementary material, which is available to authorized users.

## Graphical abstract

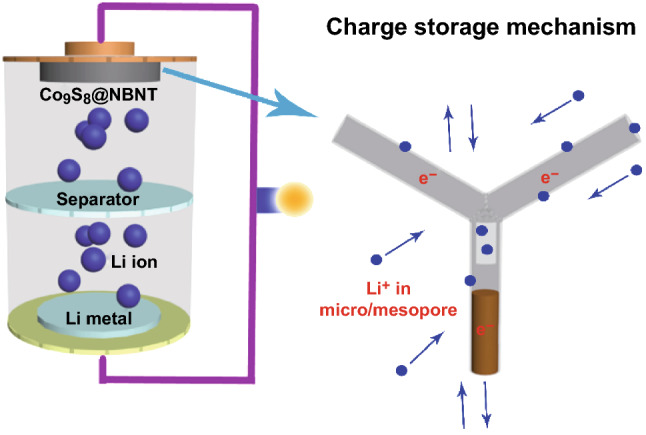

## Introduction

Recently, rechargeable energy-storage systems have attracted a great deal of interest because of the escalating requirements of various applications such as hybrid electric vehicles and electronic devices [1–4]. Among various alternatives, lithium-ion batteries (LIBs) have attracted unprecedented attention owing to increasing market demand [5–8]. However, LIBs suffer from a lack of high-performance anode materials, which hinders their practical application [7–11]. Extensive studies have been conducted to solve these problems by structure design to achieve different charge-storage mechanisms [10, 11]. Recently, various transition metal sulfides have been proposed because of their high theoretical capacities [12–17]. However, they are limited by their poor rate performance and sharp capacity fading caused by their low electronic conductivity and large volume changes during the charging/discharging process [9, 10, 18]. In recent years, nanostructured carbonaceous materials have been widely investigated to overcome these low capacity and kinetic limitations [18]. Carbon nanotubes (CNTs), one of the promising nanostructured carbonaceous materials with excellent electrical conductivity, large specific surface area, electrochemical and thermal stabilities, and easy ion accessibility, are expected to be an important option for LIBs [19–21]. In particular, Li^+^ ions can be intercalated not only into the intertube channel, but also into the inner space of the tube cavity, leading to excellent rate performance [22]. However, using CNTs as active materials is difficult because of their limited capacity [23–26].

Therefore, much work has been carried out to design new nanostructured hybrids for the construction of transition metal sulfide/carbon-based material composites as next-generation anodes [27–32]. One-dimensional (1D) nanomaterials, which have a large accessible area, fast ion diffusion, and percolated electron transport, are considered to be ideal nanoscale building blocks for construction of multidimensional and multifunctional electrode configurations for advanced electrochemical energy storage [27, 33–35]. Hence, construction of integrated 1D nanostructured transition metal sulfides with CNTs can also be regarded as an attractive strategy for developing high-performance anode materials for LIBs. Although high-capacity transition metal sulfides have been incorporated onto the surface of hierarchical CNT networks to improve the rate and cycling performances, these active materials are still unstable because of surface exposure and interparticle aggregation [24].

**Fig. 1:**
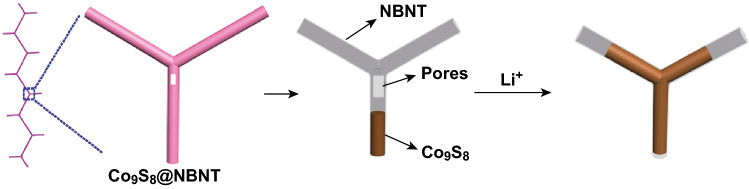
Illustration of lithium-ion storage in a Co_9_S_8_@NBNT electrode

Herein, we demonstrate a unique hierarchical hybrid architecture of Co_9_S_8_@NBNT for LIBs, wherein cobalt sulfide nanowires are encapsulated inside N-doped porous branched carbon nanotubes (NBNTs) (Fig. 1). Such a unique electrode configuration is expected to have the following features: (1) The 3D networks of NBNTs significantly enhance the electronic conductivity of the hybrid, promoting Li^+^ diffusion and electron transport through the 3D interconnected pathway; and (2) the NBNT branches will inhibit the volume expansion of the encapsulated cobalt sulfide during the charge/discharge processes so as to maintain the structural stability.

## Experimental

### Materials Synthesis

The Co_9_S_8_@NBNT was synthesized by a catalytic decomposition reaction of polyacrylonitrile (PAN) on a Co/MgO catalyst in the presence of sodium polysulfide. The Co/MgO catalyst was prepared as follows. Co(NO_3_)_2_·6H_2_O and Mg(NO_3_)_2_·6H_2_O were mechanically mixed and ground, and then calcined at 600 °C for 1 h in air to decompose the precursor and yield a cluster made of Co and Mg oxides. The formed powder was then reduced in H_2_ (100 sccm) and Ar (200 sccm) for 30 min at 600 °C to form Co nanoclusters supported on MgO particles, which were collected and used as the catalyst.

A sodium polysulfide (Na–poly-S) solution was prepared from sulfur (6 g, Puriss, precipitated, 99.5–100.5%) and sodium disulfide (nonahydrate, 18 g, ACS reagent 98%) in water (240 g, distilled) by sonication and stirring overnight with mild heating (40 °C). l-Ascorbic acid (80 mg, 99%) was dissolved in water (26 g, distilled) in a 40 mL glass vial with a PET cap. Subsequently, 4 g of the Na–poly-S solution was added and hand-shaken. Hydrochloric acid (0.1 mL, Puranal 37%, diluted to 5 M) was then added, and the vial was hand-shaken. The vial was closed, hand-shaken and sonicated at 40 °C for 30 min. The Co/MgO catalyst particles and PAN powder were placed in a graphite crucible enclosed within a graphite susceptor, and heated up to the reaction temperature using an induction furnace with a flow of Ar (2300 sccm) and H_2_ (100 sccm). H_2_ was allowed to bubble through the vial. The temperature of the susceptor was controlled to ensure that Co/MgO catalyst particles were heated to 1000 °C. After growth for 15 min, the H_2_ flow was stopped and the chamber was cooled down to room temperature. During the cooling process, the system was purged with Ar to prevent a backflow of air from the exhaust line.

The obtained product was washed three times by replacing the liquid products with distilled water and HCl, allowing soluble components to diffuse out of the product for at least 2 h. The washed product was freeze-dried to remove the liquid component. The resulting product was cut into disks with a razor blade. Prior to use as an anode, the disks were dried in a vacuum oven at 90 °C for 1 h and directly transferred to an argon-filled glove box.

Co_9_S_8_@CNT was obtained by a catalytic decomposition reaction of dimethyl sulfide (C_2_H_6_S) on the Co/MgO catalyst, which was reported in our previous work [34].

Commercial multiwalled CNTs were obtained from Shenzhen Nano Co. Ltd.

### Characterization

The products were characterized by scanning electron microscopy (SEM) and high-resolution transmission electron microscopy (HRTEM, JEM-2010). N_2_ sorption analysis was performed on an ASAP 2020 accelerated surface area and porosimetry instrument (Micromeritics) equipped with an automated surface area analyzer at 77 K, using Barrett–Emmett–Teller (BET) calculations for the surface area. The pore-size distribution (PSD) plot was prepared from the adsorption branch of the isotherm based on a density functional theory (DFT) model. X-ray powder diffraction (XRD) patterns of the sample were recorded using a D/Max-3C diffractometer equipped with a Cu-K*α* X-ray source. Raman spectra were measured using a Renishaw inVia Raman spectrometer system (Gloucestershire, UK) equipped with a Leica DMLB microscope (Wetzlar, Germany) and a 17 mW at 633 nm Renishaw helium–neon laser source. X-ray photoelectron spectroscopy (XPS) measurements were taken on a Kratos XSAM 800 spectrometer with a Mg–K*α* radiation source. TGA was carried out using a DuPont 2200 thermal analysis station.

### Electrochemical Measurements

The electrochemical tests were conducted by cycling two-electrode 2032 coin cells with Li disks as both the counter and reference electrode, a Celgard 2400 film as the separator, and a mixed slurry consisting of the prepared Co_9_S_8_@NBNT structure (80 wt%) with poly(vinylidene fluoride) (PVDF, 20 wt%) in *N*-methyl-2-pyrrolidone (NMP) without conducting agents. The Co_9_S_8_@NBNT composite electrodes were pressed before being assembled into the coin cells. The loading density, diameter, and thickness of the prepared electrodes were ~ 1 mg cm^−2^, ~ 12 mm, and ~ 65–85 μm, respectively. The electrolyte was 1 M LiPF_6_ in a 50:50 (w/w) mixture of ethylene carbonate and diethyl carbonate. Cyclic voltammetry and electrochemical impedance spectroscopy were conducted with a CHI 660C electrochemical workstation.

## Results and Discussion

**Fig. 2:**
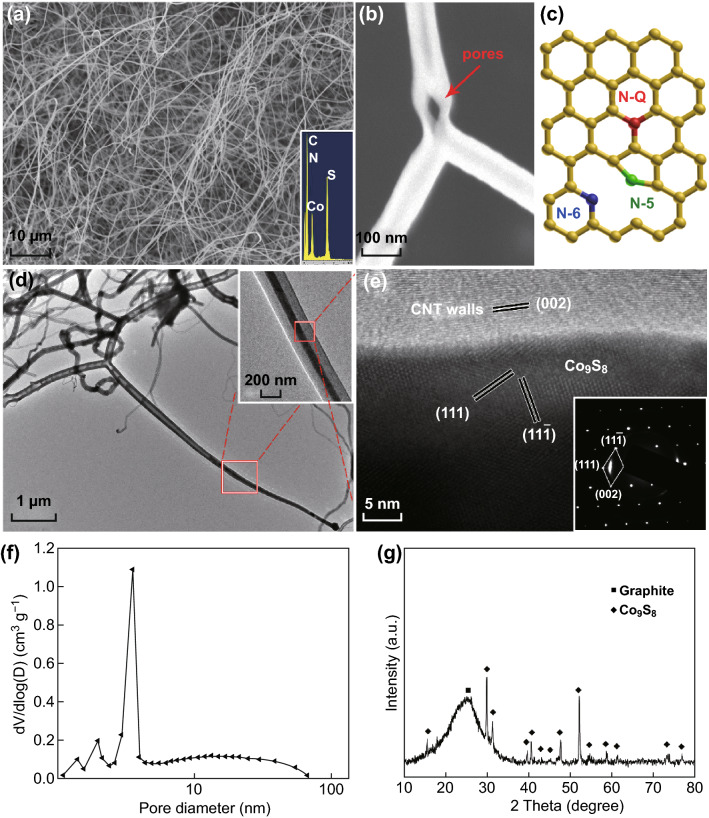
**a** SEM image and corresponding EDX spectra of Co_9_S_8_@NBNT. **b** High-magnification SEM images of Co_9_S_8_@NBNT. The porous structures are indicated by arrows. **c** Schematic diagram of N-doped CNTs. **d** TEM image of Co_9_S_8_@NBNT. Inset is HRTEM image of the boxed area. **e** Enlargement of the boxed area shown in **d** and its corresponding SAED pattern (inset). **f** Pore-size distributions. **g** XRD spectra of Co_9_S_8_@NBNT

The morphology of the Co_9_S_8_@NBNT nanocomposites is shown in Fig. 2a. Energy-dispersive X-ray (EDX) spectroscopy firmly demonstrated the existence of C, Co, S, and N (possible locations for N incorporation into the CNTs are shown in Fig. 2c). Figures 2b and S1 show high-magnification scanning electron microscopy images of Co_9_S_8_@NBNT, with the porous structures indicated by arrows. Figure 2d contains TEM image of the Co_9_S_8_@NBNT, and inset shows HRTEM image of the boxed area; the porous structure and filled nanowires are clearly shown. Figure 2e shows an enlargement of the boxed area shown in the inset of Fig. 2d. It is clearly shown that the nanowires encapsulated in the NBNTs consisted of well-crystallized Co_9_S_8_ cores with a unit cell parameter of *a* = 0.9907 nm (JCPDS No. 75-2023). The multilayered carbon sheath exhibited an interlayer spacing of 0.34 nm, which corresponds well with the interplanar distance of the (001) planes of graphite. The selected-area electron diffraction (SAED) pattern shows the sharp spots that could be indexed as the reflections of cubic Co_9_S_8_ with the [1, − 1, 0] axis parallel to the electron beam. Remarkably, the specific surface area of Co_9_S_8_@NBNT could reach up to 985 m^2^ g^−1^ (Fig. S2). The corresponding PSD, estimated from the adsorption branches of the isotherms based on a DFT model, clearly shows the presence of multiple porosities as small as a few nanometers (Fig. 2f). As shown in Fig. 2g, the distinct diffraction peaks in XRD pattern can be assigned to cobalt sulfide (JCPDS No. 75-2023) and graphite. The as-obtained Co_9_S_8_@NBNT was also characterized by Raman spectroscopy (Fig. S3) and XPS (Fig. S4). The N 1*s* band of Co_9_S_8_@NBNT can be deconvoluted into three characteristic peaks: pyridinic, pyrrolic, and graphitic N species located at 398.6, 400.8, and 401.3 eV, respectively. The chemical composition of the NBNTs was estimated as 41.09 wt% Co_9_S_8_@NBNT, as determined by TGA analysis (Fig. S5).

**Fig. 3:**
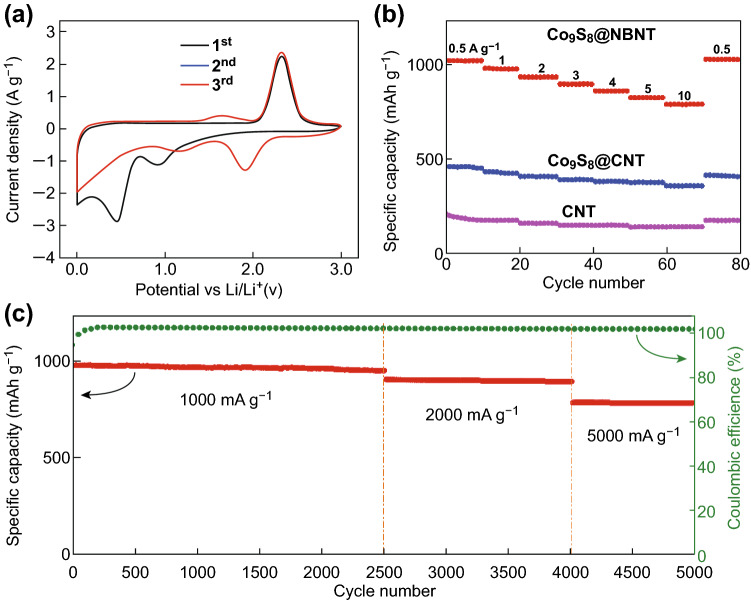
**a** CV curves of the LIBs with Co_9_S_8_@NBNT. **b** Rate performance at different current densities of Co_9_S_8_@NBNT, Co_9_S_8_@CNT, and CNT. **c** Long-term cycling performances of the Co_9_S_8_@NBNT anode at different current densities of 1000, 2000, and 5000 mA g^−1^

The lithium-storage properties of the Co_9_S_8_@NBNT nanohybrid were evaluated by conducting various electrochemical measurements. Figure 3a shows the first three CV curves of the Co_9_S_8_@NBNT electrode in the potential range of 0–3.0 V versus Li at a scanning rate of 0.5 mV s^−1^. During the first scan, a strong peak was observed at ~ 1.9 V, which can be attributed to Li insertion into the Co_9_S_8_ lattice to form cubic Li_2_Co_9_S_8_, as illustrated by the following reaction: Co_9_S_8_ + 2Li^+^ + 2e^−^ → Li_2_(Co_9_S_8_). Another peak close to 1.3 V was also observed in the CV curves, corresponding to the formation of Co and Li_2_S following the reaction: Li_2_(Co_9_S_8_) + 14Li^+^ + 14e^−^ → 9Co + 8Li_2_S. During charging (Li extraction), a pronounced peak at around 2.3 V can be found, which was attributed to the reconversion reaction of Co with Li_2_S to reform Co_9_S_8_. In the subsequent cycle onward, it is important to note that the CV curves almost overlapped, indicating the stable and superior reversibility of the prepared Co_9_S_8_@NBNT. Typical galvanostatic charge/discharge (GCD) curves of the nanohybrid are in agreement with the above CV curves (Fig. S6). The first reversible capacity of the Co_9_S_8_@NBNT nanohybrid is 1310 mAh g^−1^ at a current density of 0.1 A g^−1^. The rate capabilities of the Co_9_S_8_@NBNT, Co_9_S_8_@CNT, and CNT samples are presented in Fig. 3b. The Co_9_S_8_@NBNT nanohybrid electrode delivers a high reversible capacity of ~ 1109 mAh g^−1^ at a current density of 0.5 A g^−1^ with no significant decay of capacity after 200 cycles, demonstrating excellent reversibility. The capacity retention of Co_9_S_8_@NBNT was greater than those of the Co_9_S_8_@CNT and CNT samples at 500 mA g^−1^, as shown in Fig. S7. To further confirm this benefit, EIS measurements were taken at the initial state and after being charged/discharged for 200 cycles (Fig. S8). The Nyquist plots of the Co_9_S_8_@NBNT, Co_9_S_8_@CNT, and CNT samples all show a straight line in the low-frequency region, indicating Warburg-type resistance caused by ion diffusion in the electrode, and an arc in the high-frequency region. The diameter of the arc represents the charge-transfer resistance (*R*_t_). Compared with the Co_9_S_8_@CNT and CNT composites, the Co_9_S_8_@NBNT composite shows a lower *R*_t_, which demonstrates that the Co_9_S_8_@NBNT composite has much smaller interfacial charge-transfer and lithium-ion-diffusion resistances than that of the Co_9_S_8_@CNT and CNT anodes.

The outstanding cycling capability of Co_9_S_8_@NBNT at various current densities shown in Fig. 3c further proves its remarkable reversibility and stability. The capacity of the Co_9_S_8_@NBNT electrode reached 945 mAh g^−1^ after the first 2500 cycles at 1000 mA g^−1^, 889 mAh g^−1^ at 2000 mA g^−1^ after 1500 cycles, and 779 mAh g^−1^ after another 1000 cycles at 5000 mA g^−1^, respectively. The coulombic efficiency of the Co_9_S_8_@NBNT electrode was nearly 100% after the first few cycles. The irreversible capacity loss in the first cycle can mainly be attributed to the incomplete extraction of Li ions as a result of lithium being trapped in the porous electrodes and unable to be released in the first cycle, the irreversible formation of a solid-electrolyte interface layer, and the formation of insulating Li_2_S, which is generally observed for nanostructured conversion-based anode materials [35, 36]. This result is consistent with the CV results in which the cathodic peaks only exist in the first cycle and are absent in subsequent cycles.

**Fig. 4:**
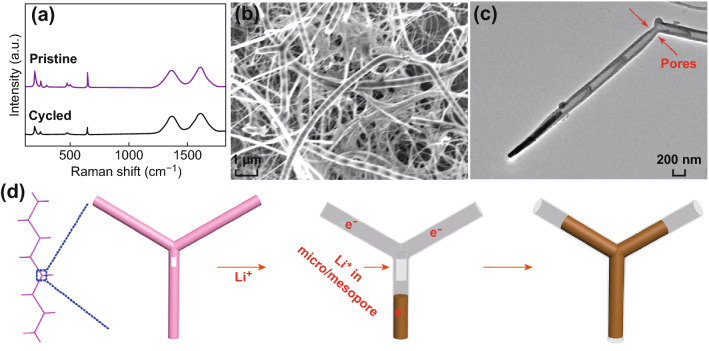
**a** Raman spectra of the pristine and cycled electrode. **b** SEM and **c** TEM images of Co_9_S_8_@NBNT after 200 cycles. **d** Schematic illustration of electron transfer and lithium-ion storage in the Co_9_S_8_@NBNT electrode

In order to confirm the structural integrity associated with the cycling stability, we carried out a postmortem study using Raman, field-emission SEM, and TEM examinations. Raman spectra of the pristine and cycled electrodes are shown in Fig. 4a. The active material in the electrode was well maintained after long-term cycling. Figure 4b shows a SEM image of the Co_9_S_8_@NBNT nanohybrid after cycling. By comparison with the fresh electrode (Fig. 2a), it can be seen that the hierarchical structure of the Co_9_S_8_@NBNT electrode was preserved after 200 charging/discharging cycles. The cobalt sulfide in the darker color was still encapsulated within the NBNTs, as can be seen in the TEM image (Fig. 4c). Moreover, the porous structures were preserved (indicated by arrows in Figs. 4c and S9). As shown in Fig. 4d, the active cobalt sulfide nanowires could expand slightly in the length direction, while expansion in the width direction was prevented by the NBNT wall. Clearly, the size, shape, and structural integrity the 1D Co_9_S_8_@NBNT were well retained. These results demonstrate the great structural advantages of such structures. Firstly, the volume expansion and mechanical stress can be relieved during cycling by expansion along the available void space of the tube (Fig. 4d). Secondly, the porous structure plus the high specific surface area (Figs. 2f and S1) of Co_9_S_8_@NBNT nanohybrid can shorten the ion-diffusion distance by providing 3D interconnected NBNT networks and conducting pathways of the CNT branches to facilitate electron transport. When the lithium ions were transported through the interconnected porous channels, they could penetrate into the tube interiors through the micro/mesopores (Fig. 4d). Thirdly, the pyridinic and pyrrolic nitrogen (Fig. S4) located at defects were beneficial for adsorption of Li and provided additional Li storage sites [37–39].

## Conclusions

In summary, we have designed and prepared a novel hierarchical structure constructed by encapsulating cobalt sulfide nanowires within nitrogen-doped porous branched carbon nanotubes. The Co_9_S_8_@NBNT nanohybrid exhibited remarkable electrochemical performance as an anode material in LIBs with a very high specific capacity of up to 1310 mAh g^−1^ at a current density of 0.1 A g^−1^, outstanding rate capability, and long cycle life. The Co_9_S_8_@NBNT with hierarchical porosity, the incorporation of nitrogen doping, and interconnected NBNT networks played a role in improving the rate capability by allowing rapid Li^+^ diffusion and facilitating electronic transport. The CNT-confinement of the active cobalt sulfide nanowires offered a proximate electron pathway for the isolated nanoparticles and shielding of the cobalt sulfide nanowires from pulverization for long cycling time periods. Such a strategy could be readily extended to other materials for energy-storage and conversion applications.

## Electronic supplementary material

Below is the link to the electronic supplementary material.

Supplementary material 1 (PDF 495 kb)
