# Supplementary material for: Cobalt Sulfide Confined in N-Doped Porous Branched Carbon Nanotubes for Lithium-Ion Batteries
Source: Nanomicro Lett. 2019 Mar 29;11:29. doi: 10.1007/s40820-019-0259-z (PMC7770667; doi:10.1007/s40820-019-0259-z)
Supplement: Supplementary file 1 — Supplementary material 1 (PDF 495 kb) [file 40820_2019_259_MOESM1_ESM.pdf]

Supporting Information for

Cobalt Sulfide Confined in N-doped Porous Branched Carbon Nanotube for Lithium-Ion Batteries

Yongsheng Zhou[1, 2, *], Yingchun Zhu[2], Bingshe Xu[3], Xueji Zhang[4, *], Khalid A. Al-Ghanim[5], Shahid Mahboob[5, 6]

[1]College of Chemistry and Materials Engineering, Anhui Science and Technology University, Bengbu 233030, People's Republic of China

[2]Key Laboratory of Inorganic Coating Materials CAS, Shanghai Institute of Ceramics, Chinese Academy of Sciences, Shanghai 200050, People's Republic of China

[3]Key Laboratory of Interface Science and Engineering in Advanced Materials, Ministry of Education, Taiyuan University of Technology, Taiyuan 030024, People's Republic of China

[4]Beijing Key Laboratory for Bioengineering and Sensing Technology, Research Center for Bioengineering and Sensing Technology, School of Chemistry and Bioengineering, University of Science & Technology Beijing, 30 Xueyuan Road, Haidian District, Beijing 100083, People's Republic of China

[5]Department of Zoology, College of Science, King Saud University, Riyadh, Saudi Arabia

[6]Department of Zoology, GC University, Faisalabad, Pakista

*Corresponding authors. E-mail: yszhou1981@gmail.com (Yongsheng Zhou); zhangxueji@ustb.edu.cn (Xueji Zhang)

Supplementary Figures and Table

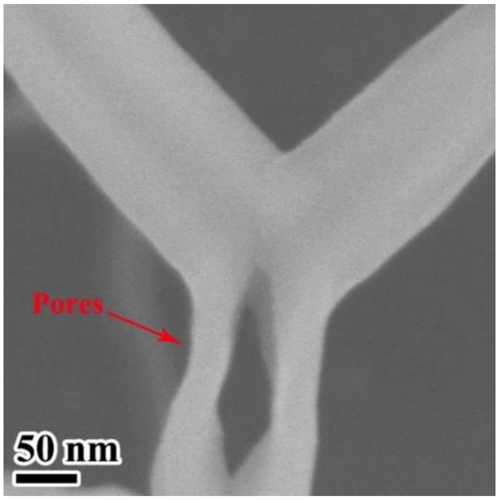

Fig. S1 High-magnification SEM image of Co_9S_8@NBNT

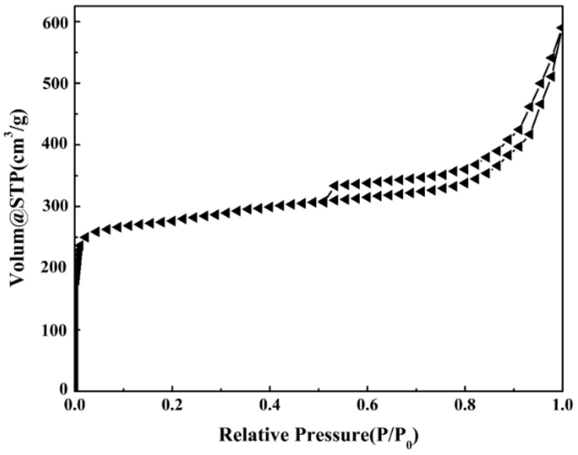

Fig. S2 N_2 sorption isotherms of Co_9S_8@NBNT

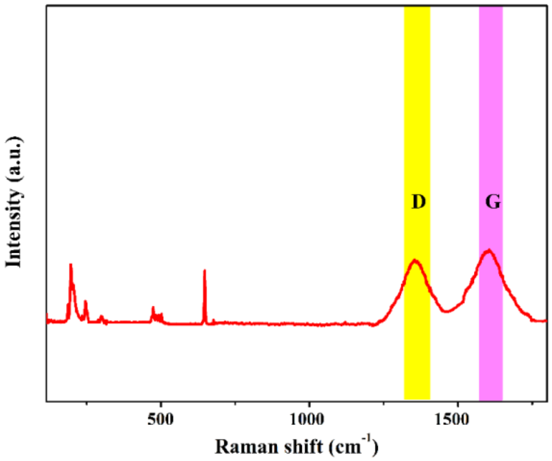

Fig. S3 Raman spectra of the Co_9S_8@NBNT. Besides the D, and G bonds, corresponding to the NBNT, there are new peaks corresponding to the Co_9S_8

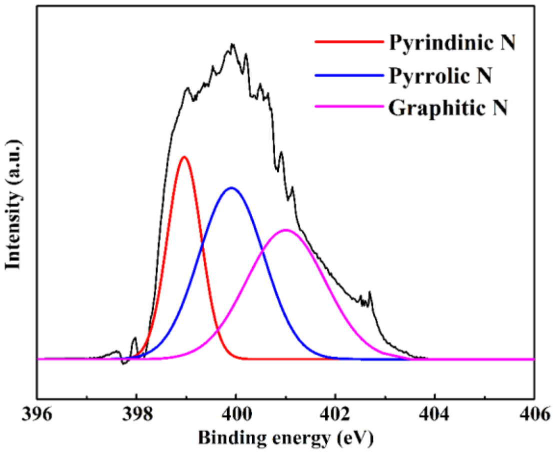

Fig. S4 High-resolution XPS N 1s spectrum of Co_9S_8@NBNT

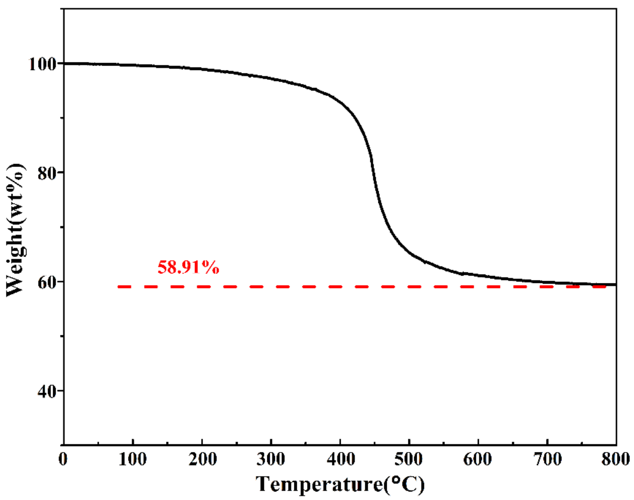

Fig. S5 TGA curve of Co_9S_8@NBNT

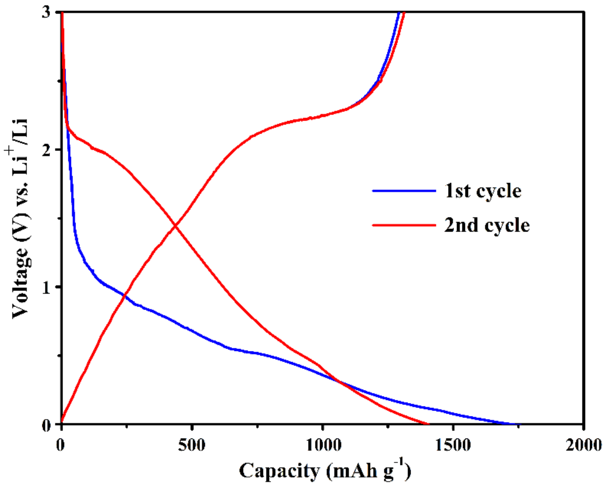

Fig. S6 Charge-discharge voltage profiles at 0.1 A g^{-1} of Co_9S_8@NBNT

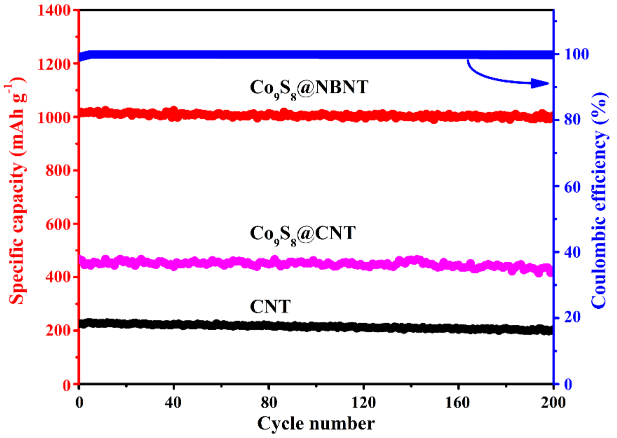

Fig. S7 Comparison of cycle stability at 500 mA g^{-1} of the obtained samples

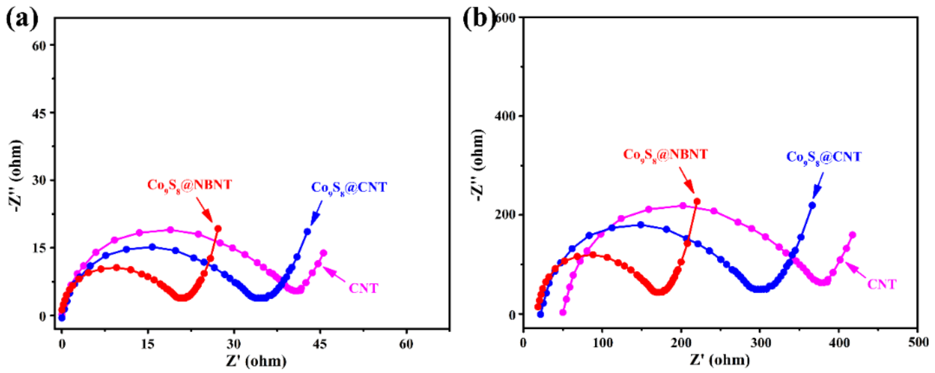

Fig. S8 Nyquist plots of Co_9S_8@NBNT, Co_9S_8@CNT, and CNT samples **a** before and **b** after cycling test

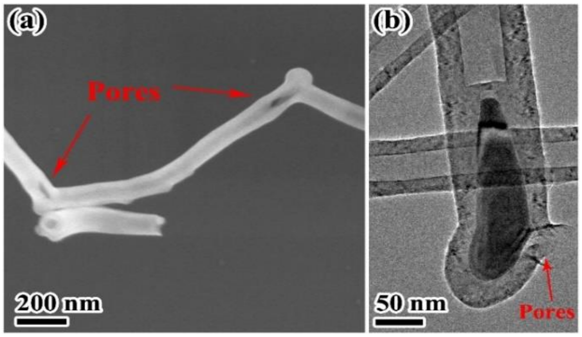

Fig. S9 a High-magnification SEM and **b** HRTEM images of Co_9S_8@NBNT after 200th cycles

Table S1| Different carbon materials employed in cathodes for LIBs

Materials	Initial capacity (mAh g^{-1})	Reversible specific capacity	References
N-C-800	3487	2132 mAh g^{-1} at 100 mA g^{-1} after 500 cycles	[S1]
CNT-on-OCNT-Fe	985	784 mAh g^{-1} at 100 mA g^{-1} after 200 cycles	[S2]
HN-CNT	730	397 mAh g^{-1} at 100 mA g^{-1} after 100 cycles	[S3]
Sn/Sn_4P_3@C	1332	589 mAh g^{-1} at 1000 mA g^{-1} after 700 cycles	[S4]
$CuO/Cu_2O/C$	381	260 mAh g^{-1} at 200 mA g^{-1} after 600 cycles	[S5]
Si@C@MoS_2	2079.3	1365.7 mAh g^{-1} at 500 mA g^{-1} after 500 cycles	[S6]
CoSe/NC-L	530	424 mAh g^{-1} at 500 mA g^{-1} after 150 cycles	[S7]
WS_2@C	730	638 mAh g^{-1} at 500 mA g^{-1} after 45 cycles	[S8]
pSiMS@C	1665	1027.8 mAh g^{-1} at 1000 mA g^{-1} after 500 cycles	[S9]
Our work	1310	1109 mAh g^{-1} at 500 mA g^{-1} after 200 cycles	

Supplementary References

[S1] F.C. Zheng, Y. Yang, Q.W. Chen, High lithium anodic performance of highly nitrogen-doped porous carbon prepared from a metal-organic framework. Nat. commun. **5**, 5261 (2014). https://doi.org/10.1038/ncomms6261

[S2] P. Bhattacharya, D. Suh, P. Nakhanivej, Y. Kang, H. Park, Iron Oxide Nanoparticle-encapsulated CNT branches grown on 3D ozonated CNT internetworks for lithium-ion battery anodes. Adv. Funct. Mater. 1801746 (2018). https://doi.org/10.1002/adfm.201801746

[S3] X.F. Li, J. Liu, Y. Zhang, Y.L. Li, H. Liu et al., High concentration nitrogen doped carbon nanotube anodes with superior li^+ storage performance for lithium rechargeable battery application. J. Power Sources **197**, 238-245 (2012). https://doi.org/10.1016/j.jpowsour.2011.09.024

[S4] Q. Liu, J. Ye, Z. Chen, Q. Hao, C. Xu, J. Hou, Double conductivity-improved porous Sn/Sn_4P_3@carbon nanocomposite as high-performance anode in Lithium-ion batteries. J. Colloid Inter. Sci. **537**, 588-596 (2019). https://doi.org/10.1016/j.jcis.2018.11.060

[S5] C.X. Xu, K.V. Manukyan, R.A. Adams, V.G. Pol, P.W. Chen, A. Varma, One-step solution combustion synthesis of $CuO/Cu_2O/C$ anode for long cycle life Li-ion batteries. Carbon **142**, 51-59 (2019). https://doi.org/10.1016/j.carbon.2018.10.016

[S6] X.L. Zhang, L.W. Huang, P. Zeng, L. Wu, Q.Q. Shen, Z.X. Gao, Y.G. Chen, Hierarchical MoS_2 anchored on core-shell Si@C with increased active-sites and charge transfer for superior cycling and rate capability in lithium-ion batteries. Chem. Eng. J. **357**, 625-632 (2019). https://doi.org/10.1016/j.cej.2018.09.163

[S7] X. D. Li, W. D. Zhang, Y. Feng, W. Li, P. Peng et al., Ultrafine CoSe nano-crystallites confined in leaf-like N-doped carbon for long-cyclic and fast sodium ion storage. Electrochim. Acta **294**, 173-182 (2019). https://doi.org/10.1016/j.electacta.2018.10.012

[S8] J.Q. Zhang, H. Sade, Y.F. Zhao, A.T. Murdock, A. Bendavid, J.P. Lellouche, G.X. Wang, Z.J. Han, Conformal carbon coating on WS_2 nanotubes for excellent electrochemical performance of lithium-ion batteries. Nanotechnology **30**, 035401(2019). https://doi.org/10.1088/1361-6528/aaec4d

[S9] K. Wang, S.E. Pei, Z.S. He, L.A. Huang, S.S. Zhu, J.F. Guo, H.B. Shao, J.M. Wang, Synthesis of a novel porous silicon microsphere@carbon core-shell composite via in situ MOF coating for lithium ion battery anodes. Chem. Eng. J. **356**, 272-281 (2019). https://doi.org/10.1016/j.cej.2018.09.027
